# Analyzing resistome in soil and Human gut: a study on the characterization and risk evaluation of antimicrobial peptide resistance

**DOI:** 10.3389/fmicb.2024.1352531

**Published:** 2024-03-25

**Authors:** Chongyi Zhao, Shuo Yan, Ying Luo, Yuzhu Song, Xueshan Xia

**Affiliations:** Faculty of Life Science and Technology, Kunming University of Science and Technology, Kunming, China

**Keywords:** antimicrobial peptide, antimicrobial resistance, cross-resistance, human gut, metagenomics, resistome, soil

## Abstract

**Objective:**

The limited existing knowledge regarding resistance to antimicrobial peptides (AMPs) is hindering their broad utilization. The aim of this study is to enhance the understanding of AMP resistance, a pivotal factor in the exploration of alternative drug development in response to the escalating challenge of antibiotic resistance.

**Methods:**

We utilized metagenomic functional selection to analyze genes resistant to AMPs, with a specific focus on the microbiota in soil and the human gut. Through a combination of experimental methods and bioinformatics analyses, our investigation delved into the possibilities of the evolution of resistance to AMPs, as well as the transfer or interchange of resistance genes among the environment, the human body, and pathogens. Additionally, we examined the cross-resistance between AMPs and evaluated interactions among AMPs and conventional antibiotics.

**Results:**

The presence of AMP resistance, including various resistance mechanisms, was observed in both soil and the human gut microbiota, as indicated by our findings. Significantly, the study underscored the facile evolution of AMP resistance and the potential for gene sharing or exchange among different environments. Notably, cross-resistance among AMPs was identified as a phenomenon, while cross-resistance between AMPs and antibiotics was found to be relatively infrequent.

**Conclusion:**

The results of our study highlight the significance of taking a cautious stance when considering the extensive application of AMPs. It is imperative to thoroughly assess potential resistance risks, with a particular focus on the development of resistance to AMPs across diverse domains. A comprehensive grasp of these aspects is essential for making well-informed decisions and ensuring the responsible utilization of AMPs in the ongoing fight against antibiotic resistance.

## 1 Introduction

Antimicrobial resistance (AMR) poses a significant risk to human health and has become a worldwide issue within the healthcare sector. In 2019, approximately 4.95 million deaths were linked to bacterial resistance, with 1.27 million directly linked to AMR (Antimicrobial Resistance Collaborators, 2022). The rise of resistance to traditional antibiotics has spurred efforts to discover novel antimicrobial agents.

Antimicrobial peptides (AMPs), present in nearly all living organisms, stand out as highly promising agents due to their extensive and rapid antimicrobial capabilities. The primary mode of action for AMPs involves the destruction of bacteria, primarily through membranolytic effects and other intracellular targeting mechanisms. These mechanisms include binding to nucleic acids and proteins, influencing cell cycles, and disrupting energy metabolism (Scocchi et al., 2016). Numerous AMPs have demonstrated their efficacy in inhibiting antibiotic-resistant microorganisms *in vivo*, and they can be employed either independently or in conjunction with traditional antibiotics or other antimicrobial agents to achieve synergistic effects (Sheard et al., 2019). Beyond their bactericidal impact, AMPs in higher organisms also display immunomodulatory and anti-inflammatory effects (Hancock et al., 2016).

These versatile attributes of AMPs have fueled increased interest in the biopharmaceutical industry, leading to significant investments in the AMPs market. The Global Antimicrobial Peptides market, valued at USD 5 million in 2020, is anticipated to reach USD 6 million by the close of 2027, with a compound annual growth rate (CAGR) of 5.4% from 2022 to 2027 (Global Antimicrobial Peptides Sales Market Report, 2021). Currently, more than 400 peptides are undergoing clinical phase trials, and over 60 peptides have received approval from the US Food and Drug Administration (FDA) (Agarwal and Gabrani, 2021). However, despite the substantial investment in peptide therapies for metabolic diseases like obesity, interest in peptide-based antibiotics within the biopharmaceutical industry remains limited. This discrepancy underscores a potential gap in research and investment priorities, with antibiotic development potentially being overshadowed by other therapeutic areas with higher profit margins.

Moreover, advancements in AMP design, peptide synthesis, and biotechnology have exhibited remarkable potential in overcoming the stability, toxicity, and activity limitations associated with natural AMPs. Consequently, the utilization of AMPs is expected to witness a substantial increase in various fields, including food preservation, agriculture, the environment, animal husbandry, and the pharmaceutical industry.

A subsequent issue pertains to the emergence and spread of resistance to AMPs. Despite initial assertions suggesting that AMPs might not provoke resistance, subsequent research has revealed that AMP resistance could be as concerning as resistance to traditional antibiotics. AMP resistance can emerge under selective pressures and be acquired through horizontal transfer (Arcilla et al., 2016). Furthermore, resistance to an AMP may compromise the efficacy of other AMPs and impact immunoregulatory functions in humans (Joo et al., 2016; Blanco et al., 2020). These factors pose limitations on the widespread use of AMPs across various domains. Currently, the risks associated with AMP resistance have received insufficient attention and lack systematic assessment, particularly in comprehensively analyzing resistance characteristics from environmental, human, and pathogenic perspectives. Ultimately, the evolution of resistance to antimicrobials in the natural environment may translate into resistance against clinical antibiotics (Colclough et al., 2019).

As representatives of the natural environment and the human body, both soil and the human gut are viewed as repositories of resistance against traditional antibiotics. Utilizing metagenomic functional selection and advanced sequencing methods, we examined the resistome of AMPs in these ecosystems. Through this analysis, we sought to outline essential aspects of AMP resistance, such as the potential for its rapid evolution, the underlying mechanisms, the likelihood of resistance transmission, and the prevalence of cross-resistance. The objective of this study is to offer a thorough evaluation of the risks linked to AMP resistance.

## 2 Materials and methods

### 2.1 Creating a comprehensive known AMP resistance gene data set

We conducted a literature search using the keywords ‘antimicrobial peptide’ and ‘resistance’ on PubMed NCBI and Google Scholar. Our curated list of genes related to antimicrobial peptide resistance included those identified by Kintses et al. (2019), comprising 138 genes. Additionally, our search contributed an extra 298 genes, resulting in a total of 436 antimicrobial peptide resistance genes in our compiled list (Supplementary Table S1).

### 2.2 Antimicrobial peptides

Synthesized AMPs such as Iseganan (IB), Melittin (GQ) and Cathelicidin-DM (SA) were procured from Dangang Biotechnology Company. Polymyxin B (PB) and colistin (CST) were acquired from Solarbio. Specific information about these five AMPs are shown in Table 1.

**Table 1:** Detailed information of the 5 antimicrobial peptides used in this study.

AMP	Origin	Length (aa)	3D structure	Practical applications	Abbreviation in this article
Melittin	Bee venom	26	α-helix	Model AMP, no practical use	GQ
Iseganan	Porcine leukocytes	18	β-fold	Phase III clinical trial for the treatment of oral mucositis after radiotherapy for head and neck cancer	IB
Cathelicidin-DM	*Duttaphrynus melanostictus*	37	Unknown	None	SA
Polymyxin B	*Bacillus polymyxa*	10	Unknown	Clinically used in multidrug-resistant gram-negative bacterial infections	PB
Colistin	*Bacillus polymyxa*	10	Unknown	It was widely used in animal husbandry. It is now used for clinically multidrug-resistant gram-negative bacterial infections and veterinary	CST

### 2.3 Bacterial strains and culturing conditions

*Escherichia coli* DH5α, *E. coli* BL21(DE3), and *E. coli* JM109 were utilized in this study. LB agar (10 g/L tryptone, 5 g/L yeast extract, 10 g/L NaCl and 15 g/L agar) and LB broth were employed in all growth trials, while Mueller Hinton Broth (MHB) without cation adjustment was utilized for minimal inhibitory concentration (MIC) testing.

### 2.4 Preparation of electrocompetent and chemically competent cells

Electrocompetent cells of *E. coli* DH5α were generated, while chemically competent cells of *E. coli* BL21(DE3) and *E. coli* JM109 were also prepared (Renzette, 2011).

### 2.5 MIC determination for five antimicrobial peptides

The MICs were determined using the broth microdilution method in 96-well microtiter plates (Wiegand et al., 2008). In brief, a 1:100 dilution of the mid-exponential phase bacterial culture (OD600 = 0.5), consisting of 5 μL (1 × 10^5^ CFU/mL), was introduced into polypropylene 96-well plates. These plates contained a two-fold dilution series of AMP in a total volume of 100 μL MHB per well. The incubation of the plates occurred at 37°C in a humidity chamber. The MIC was identified as the lowest concentration that hindered visible bacterial growth after 24 h of incubation. Each experiment was conducted in triplicate. In our study, the MICs for *E. coli* DH5α containing pUC118 plasmids against IB, GQ, SA, PB, and CST were 16 μg/mL, 16 μg/mL, 16 μg/mL, 0.5 μg/mL, and 0.25 μg/mL, respectively.

### 2.6 Extraction and purification of soil DNA

Soil samples from various locations and at different elevations were collected, such as at Kunming University of Science and Technology, Tianyuan Campus greenhouse (E102°51′, N25°50′, 1928 meters above sea level), the flowerbed in front of the Jingyuan cafeteria (E102°51′, N24°51′, 1949 meters above sea level), the Laoyuhu Wetland in Dianchi Lake (E102°86′, N24°85′, 1928 meters above sea level), the pine roots in Liangwang Mountain Stone Village (E102°84′, N24°72′, 2,450 meters above sea level), and the farmland in Wanxichong Village (E102°52′, N24°49′, 2020 meters above sea level). They were then combined by equal weight. The DNA extraction process began immediately without storing the soil samples. Using 20 g of soil sample, DNA extraction was conducted based on the method proposed by Zhou (Zhou et al., 1996) with some adjustments. Specifically, 5 g soil sample was mixed with 13.5 mL of DNA extraction buffer (100 mM sodium EDTA [pH 8.0],100 mM Tris–HCl [pH 8.0], 1.5 M NaCl, 100 mM sodium phosphate [pH 8.0], 1% CTAB), 200 μL lysozyme (50 mg/mL, pH 8. 0) and 100 μL of proteinase K (25 mg/mL) in Oakridge tubes. The mixture was subject to a water bath for 30 min at 37°C with gentle end-over-end inversions every 10 min. Subsequently, 1.5 mL of 20% SDS was added, and the samples were incubated in a water bath at 65°C for 2 h with gentle end-over-end inversions every 20 min. The supernatants were harvested following centrifugation at 8,000 rpm for 15 min at room temperature and then transferred to 50-ml centrifuge tubes. The soil pellets underwent two additional extraction rounds involving the addition of 4.5 mL of the extraction buffer and 0.5 mL of 20% SDS. Subsequently, the sample underwent vortexing for 10 s, followed by an incubation period at 65°C lasting for 10 min, and centrifugation as previously detailed. The supernatants obtained from the three extraction cycles were combined and mixed with an equal volume of chloroform isoamyl alcohol (24:1, vol/vol). The aqueous phase was retrieved through centrifugation and precipitated with 0.6 volume of isopropanol at room temperature for 2 h. The crude nucleic acid pellet was obtained by centrifugation at 11,000 rpm for 20 min at room temperature, washed with cold 70% ethanol, and then resuspended in 1 mL sterile deionized water. Purification of the crude DNA extract was performed using gel plus minicolumns (employing agarose gel electrophoresis followed by the passage of the excised and melted gel band through ZP301 minicolumns provided by Beijing Zomanbio biotechnology). DNA quantification was carried out using a spectrophotometer, and 15 μg of purified metagenomic DNA was utilized for downstream experiments.

### 2.7 Construction of soil metagenomic library

Purified DNA underwent digestion using the *Bam*HI restriction enzyme (Takara Code No. 1010S). Fragments ranging from 1 to 3.5 kb were selectively isolated through electrophoresis on a 1% agarose gel. The corresponding gel slice was excised, and DNA extraction was carried out using a gel purification kit (Zomanbio, ZP202). The retrieved DNA was then ligated into the pUC118 *Bam*HI/BAP vector (Takara Code No. 3321). Following ligation, ethanol precipitation was performed. In this process, 3 M sodium acetate (pH 5.2) and ethanol were successively added to the ligation reaction mixture, which was then cooled at −20°C for 20 min. Subsequently, the mixture underwent centrifugation at 4°C for 30 min at maximum speed to recover DNA.

In the transformation process, 1 μL of the resulting ligation mixture was introduced into 50 μL of electrocompetent *E. coli* DH5α cells through electroporation, following the manufacturer’s instructions (Bio-rad Gene Pulser Xcell) for *E. coli*, utilizing a 1 mm electroporation cuvette. The cells were recovered in 950 μL LB medium and then incubated at 37°C with shaking at 200 rpm for 1 h. The library size was evaluated by plating 1 μL and 0.1 μL of the recovered cells on LB agar plates containing ampicillin (50 μg/mL). The insert size was estimated through gel electrophoresis of PCR products obtained by amplifying the insert from 10 randomly selected clones from the plate. The M13 primer-pair (Forward, TGTAAAACGACGGCCAGT; Reverse, CAGGAAACAGCTATGACC) was utilized for PCR, encompassing the multiple cloning sites of the pUC118 vector. The average insert size was determined to be 1.5 Kb. Multiplying this average insert size by the number of colony forming units yielded a total size of the soil metagenomic library at 1.2 Gb. The remaining recovered cell culture was added to 9 mL of LB medium containing 50 μg/mL ampicillin, grown at 37°C for 3–4 h, frozen in 20% glycerol, and stored at −80°C for subsequent functional selection tests.

### 2.8 Construction of gut metagenomic library

To create the metagenomic library of the human gut, we randomly selected stool samples from 10 Kunming University of Science and Technology students who had not used antibiotics in the past 2 years. The Ethics Committee of the First People’s Hospital of Yunnan Province approved this study, and informed consent was obtained from the individuals contributing fecal samples. Using the QIAamp Fast DNA Stool Mini Kit (QIAGEN, Catalog no. 51604), we immediately extracted DNA from the gut microbiota after sample collection, following the manufacturer’s instructions.

Next, 15 μg of metagenomic DNA was subject to digestion with the *Eco*RI restriction enzyme (Takara, Code No.1040S). Fragments ranging from 1 to 3.5 kb were selected through electrophoresis on a 1% agarose gel. The corresponding gel slice was excised, and DNA was extracted using a gel purification kit (Zomanbio, ZP202). The retrieved DNA was then ligated into the pUC118 *Eco*RI/BAP vector (Takara Code No. 3320). The subsequent procedures, including ethanol precipitation, electroporation, determination of average insert size, total library size count, and library storage, mirrored those used for constructing the soil metagenomic library. The average insert size for the gut metagenomic library was calculated to be approximately 2.0 kb, with a total library size of 1.0 Gb.

### 2.9 Functional metagenomic selections for AMP resistance

The selection process entailed employing twice the MIC of AMP. A total of 100 μL of cell culture, obtained from thawed metagenomic library stocks, was evenly distributed on LB agar plates supplemented with 50 μg/mL ampicillin and distinct AMP (IB, 32 μg/mL; GQ, 32 μg/mL; SA, 32 μg/mL; PB, 1.0 μg/mL; CST, 0.5 μg/mL). Following overnight incubation at 37°C, colonies were collected by scraping them into 2 mL LB broth. Bacterial cells were then pelleted by centrifugation at 12,000 rpm for 1 min, the supernatant was discarded, and the pellet was re-suspended in 1 mL of nuclease-free water.

### 2.10 Resistance validation in other *Escherichia coli* strains

Before washing down the colonies, we randomly selected two clones from each plate containing distinct AMP. Colony PCRs were conducted using M13 primer pairs to validate the presence of inserts and determine their sizes. The selected colonies were then subjected to sequencing. The specific details are provided in Supplementary Table S2. Following that, plasmids were isolated using the Plasmid Midiprep Kit (Zomanbio, ZP103) following the manufacturer’s guidelines. After the plasmids were successfully reintroduced into *E. coli* JM109 and *E. coli* BL 21(DE3), the MICs of the transformed strains against the AMP used for selection were tested. This testing process was carried out in triplicate.

### 2.11 Cross-resistance exerted by resistance-conferring colonies

Following validation, the colonies displaying resistance underwent additional testing via MIC assessments for the remaining four AMPs that were not employed during the selection process. This experimental procedure was carried out in triplicate.

### 2.12 Amplification of the resistance-conferring metagenomic DNA fragments

Bacterial cells obtained through functional selection were utilized in colony PCR reactions to amplify the inserts. The PCR reactions employed the M13 primer pair, with 4 μL of 10 μM primers, 25 μL of Green Taq Mix (Vazyme biotech, P131), 4 μL of bacterial suspension, and 17 μL of ddH2O, resulting in a final volume of 50 μL. DNA amplification was conducted in a thermocycler at 95°C for 10 min, followed by 30 cycles of 95°C for 15 s, 55°C for 15 s, and 72°C for 4 min. The final extension was conducted at 72°C for 7 min. Gel recovery of PCR samples was carried out using the Gel Mini Purification Kit (Zomanbio, ZP202), adhering to the manufacturer’s instructions.

### 2.13 Sequencing and functional annotation

Samples were dispatched to Annoroad corporation and subjected to sequencing via Illumina NovaSeq 6,000/Illumina HiSeq Xte. Reads exhibiting similarity to either the vector or Illumina adapters were excluded from subsequent analysis using cross_match with the specified parameters: -gap1_only -minmatch 6 -minscore 10 -gap_init −3. Following this, the reads underwent assembly using MetaSpades (v3.9.1), and the resulting contigs were saved in individual files. ORFs were predicted utilizing ORFfinder (v0.4.3), and the resultant ORFs were stored in separate files. The contig set was annotated through the BlastX algorithm of NCBI-blast+ (v2.13.0) against the UniProt_sprot database. The annotations were then exported to a tab-delimited file, and the corresponding contig sequences were saved in FASTA format.

### 2.14 Identification of known AMP resistance genes on the metagenomic contigs

An ORF was designated as a recognized AMP resistance gene when a BLASTP sequence similarity search against a carefully curated list of AMP resistance genes produced an annotation meeting the criteria of an e-value <10^−5^, identity >30%, and coverage >70% (refer to Supplementary Table S3) (Kintses et al., 2019). The parameters applied in this context are more lenient compared to those employed in identifying known antibiotic resistance genes (as discussed in the following section). This leniency is justified by our experiment, which lends additional confidence to the identification of AMP resistance.

### 2.15 Identification of known antibiotic resistance genes

**Figure 1:**
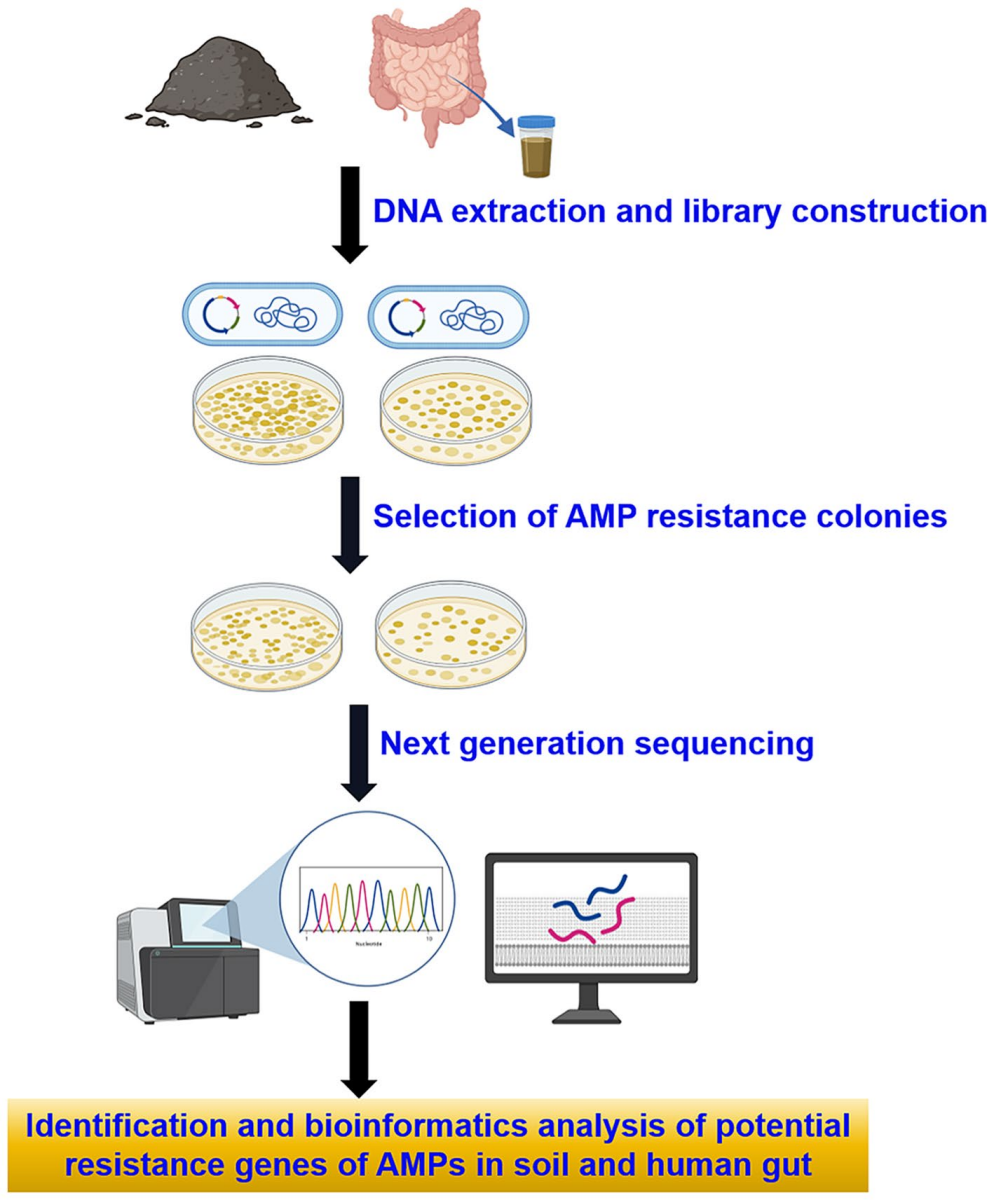
Flowchart of research methodology.

To perform functional annotation, a BLAST search was conducted against the antibiotic resistance genes found in the Comprehensive Antibiotic Resistance Database (CARD) using BLASTP, applying stringent criteria: an e-value less than 10^−5^, identity greater than 40%, and coverage exceeding 80% (Kintses et al., 2019). Subsequently, genes linked to AMP resistance were excluded, leaving behind potential genes associated with resistance to both AMPs and antibiotics (see Supplementary Table S4). The flowchart of this study is illustrated in (Figure 1).

## 3 Results

### 3.1 Human gut and soil are reservoirs of antimicrobial peptide resistance

Functional selection involved the utilization of five representative AMPs. Melittin (GQ), the principal component of bee venom, has been extensively studied for its broad spectrum of antimicrobial activities (Memariani et al., 2019). Iseganan (IB), an engineered protegrin I analog, was specifically developed for the topical treatment of oral mucositis (Koo and Seo, 2019). Cathelicidin-DM (SA), a peptide discovered in Duttaphrynus melanostictus by our laboratory, exhibits potent antibacterial effects against various pathogens (Shi et al., 2020). Polymyxin B (PB) and colistin (CST), recognized as the last line of defense against pan-drug-resistant organisms, are increasingly employed in clinical settings (Dobias et al., 2017).

**Figure 2:**
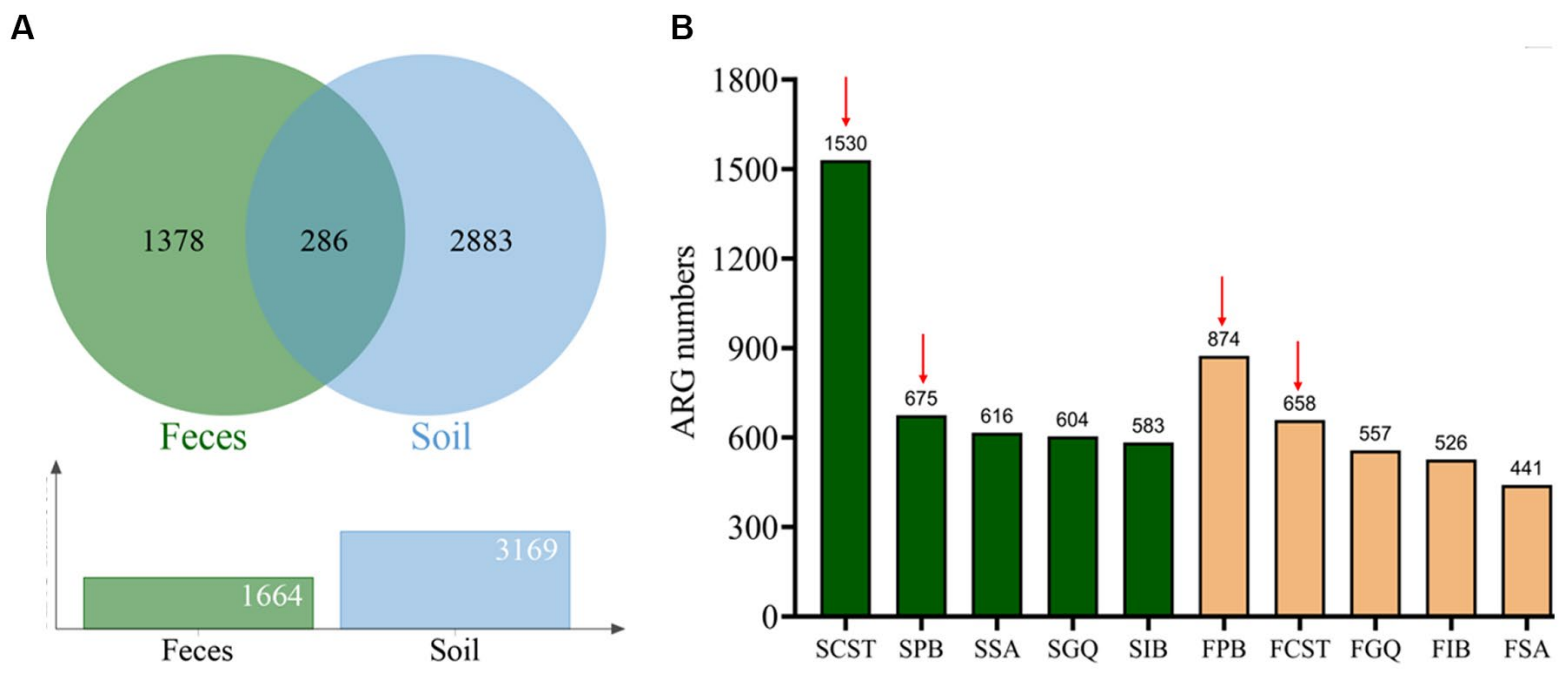
Characterization of AMP resistome in soil and human gut microbiota. **(A)** AMP resistome and shared resistance genes in the soil and human gut microbiota. **(B)** The number of resistance genes against 5 types of AMPs in the soil and human gut microbiota. Red arrows indicate the top two AMPs with the highest number of resistance genes in both samples: polymyxin B and colistin. Green columns represent resistance genes of soil microbiota, whereas yellow columns represent resistance genes of human gut microbiota.

Through metagenomic functional selection on these five AMPs, followed by next-generation sequencing technology, a total of 4,347 and 3,531 ORFs were identified from soil and human gut, respectively (refer to Supplementary Table S5). Subsequent annotation and redundancy removal using UniProt yielded a cumulative 4,547 resistance genes or resistance-related genes from soil and human gut microbiota (3,169 from soil and 1,664 from the human gut, with 286 genes overlapping; see Figure 2A). Upon comparison, the AMP resistome of soil was found to be larger than that of the human gut, with the former possessing 1.9 times the number of resistance genes compared to the latter.

In the resistome of soil, colistin resistance genes were the most prevalent, totaling 1,530 genes. Polymyxin B followed closely with 675 genes, while Cathelicidin-DM, melittin, and Iseganan had 616, 604, and 583 genes, respectively. Conversely, in the human gut resistome, polymyxin B exhibited the highest number of resistance genes at 874, with colistin at 658 genes. Melittin, Iseganan, and Cathelicidin-DM had 557, 526, and 441 genes, respectively. Figure 2B illustrates these findings, with red arrows highlighting polymyxin B and colistin as the top two antimicrobial peptides with the most resistance genes in both environments.

### 3.2 Various mechanisms are involved in AMP resistance

**Figure 3:**
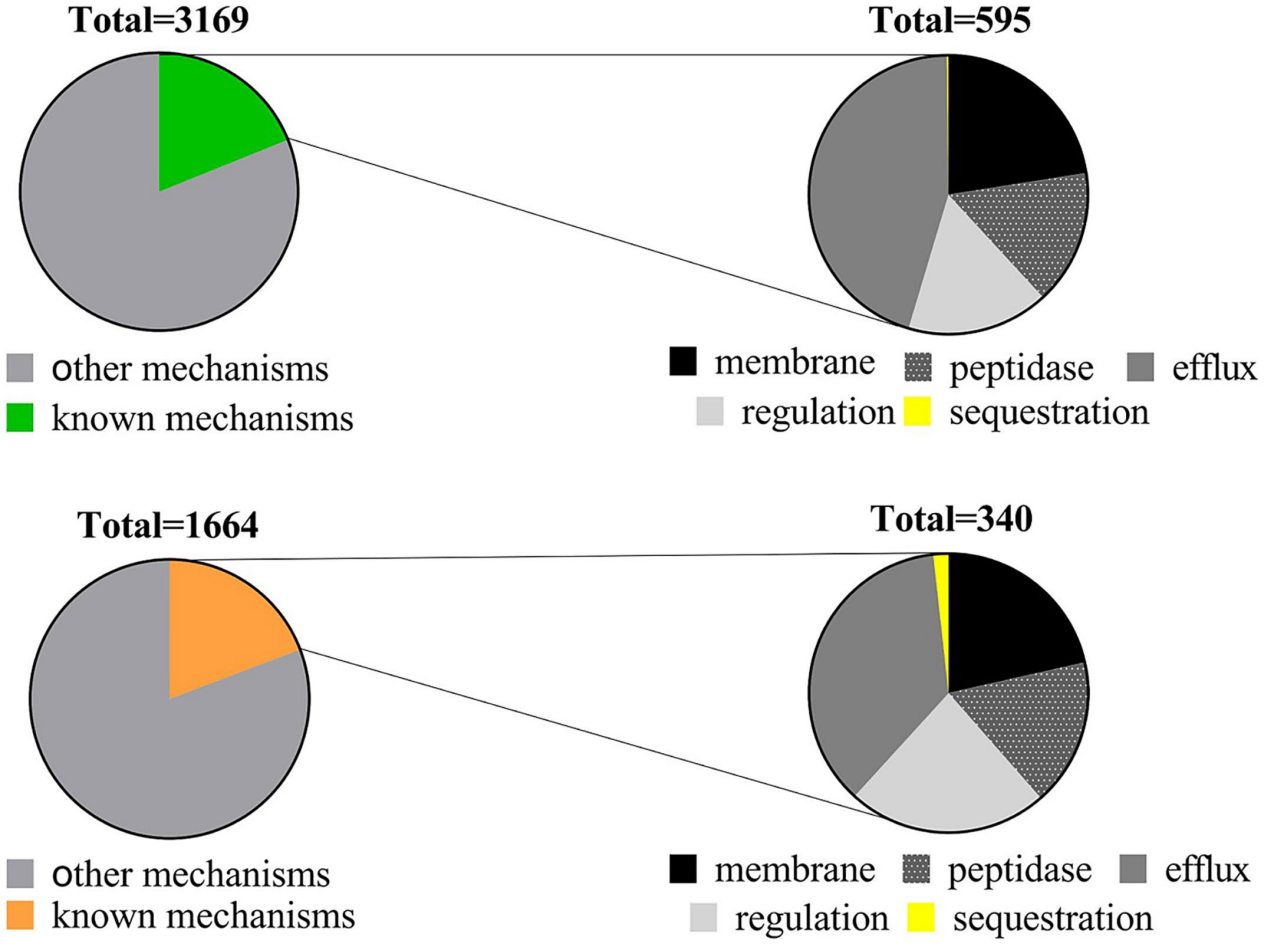
Distribution of AMP resistance mechanisms in soil (upper) and human gut (lower) microbiota.

In our examination of AMP resistance mechanisms, we categorized resistance genes into six groups: modifications in membrane/cell wall structure, protease and peptidase, regulation, transport systems and efflux pumps, sequestration, and other mechanisms (see Supplementary Table S5). Upon classifying these genes based on their mechanisms, the majority were associated with other mechanisms. The 595 resistance genes in soil and 340 in the human gut were distributed across known mechanisms. Notably, efflux pumps within the transport system were the most prevalent, followed by membrane modification, protease/peptidase, or regulatory mechanisms, while sequestration mechanisms were the least common (refer to Figure 3; Table 2). Sequestration mechanisms, which involve capturing AMPs by secreted proteins or bacterial surface structures are rare because of several factors, including the specific proteins or structures required for sequestration, variations in effectiveness across different AMPs and microbial species, and potential energy costs for microorganisms.

**Table 2:** The percentage of resistance genes for known AMPs resistance mechanisms in soil and human gut microbiota.

Mechanisms	Percentage of resistance genes	
	Soil	Human gut
Efflux pumps	45.2%	36.5%
Membrane modification	22.5%	21.5%
Protease/peptidase	15.6%	17.1%
Regulation	16.5%	23.2%
Sequestration	0.2%	1.8%

### 3.3 Levels of AMP resistance are generally low in soil and human gut

When assessing resistance, we selected two colonies at random from each plate with unique AMP content. We then extracted plasmids containing inserts, retransformed them into both *E. coli* JM109 and *E. coli* BL 21 (DE3), and subjected all clones to MIC testing against the originally used AMP (refer to the Method section). The MIC values for the colonies closely matched the concentration employed for selection, specifically, being twice the MIC of the control. Notably, in this study, the highest resistance level tested was four times the MIC of the control, as detailed in Supplementary Table S6.

### 3.4 Resistance is shared between soil, human gut, and pathogens

By comparing a dataset of acknowledged AMP resistance genes manually curated for accuracy, we pinpointed 119 and 75 AMP resistance genes within the soil and human gut microbiota, respectively. Among these, 48 genes were identified as common to both the soil and human gut samples. After eliminating duplications, a total of 146 established resistance genes were uncovered in the resistomes of both soil and the human gut. Notably, 109 of these genes were traced back to pathogenic bacteria, while 37 were linked to non-pathogenic bacteria (refer to Supplementary Table S7). This implies the potential existence of shared or mobile antimicrobial peptide-resistant bacteria and resistance genes across soil, the human gut, and pathogens.

**Figure 4:**
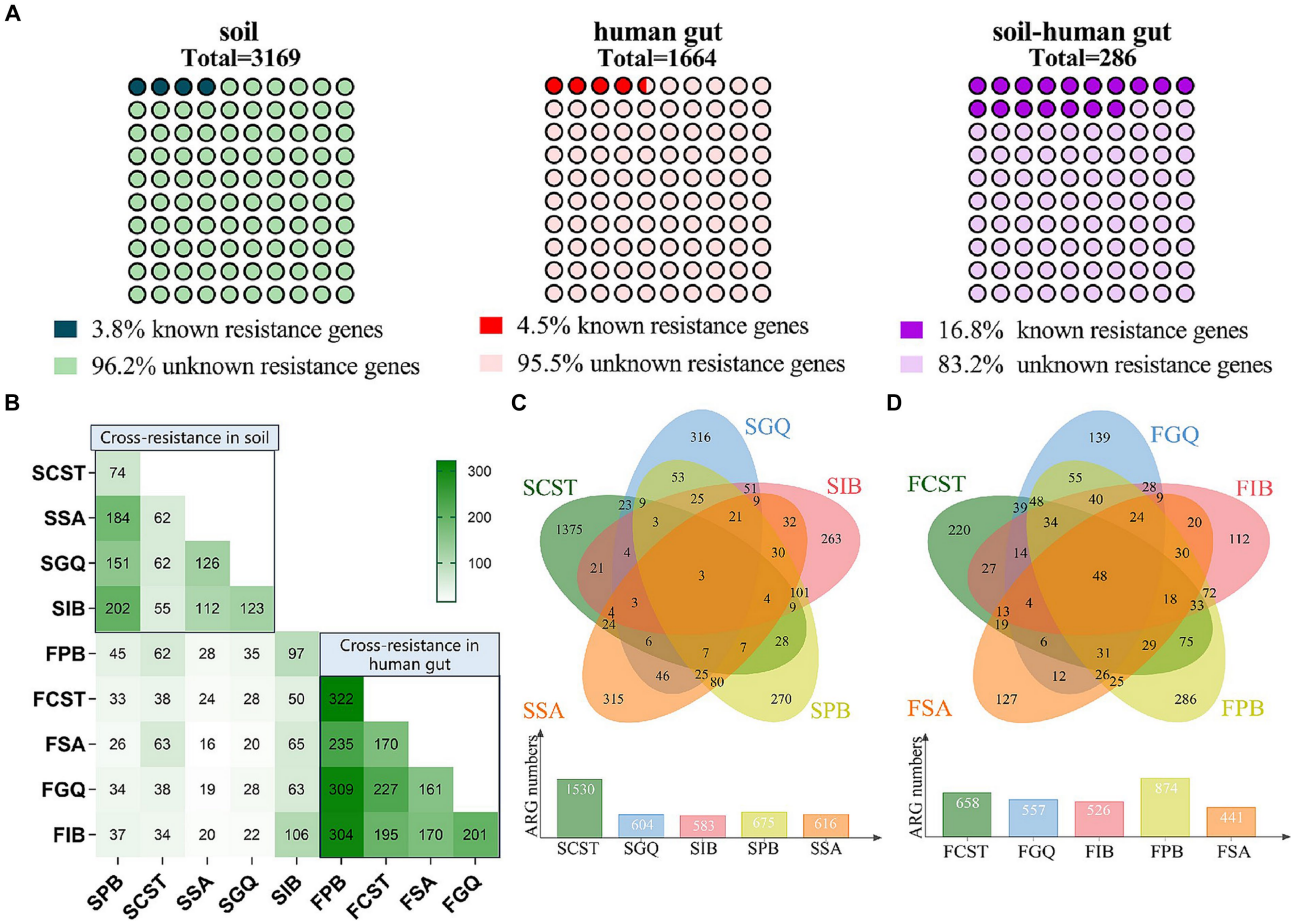
**(A)** Distribution of known and unknown resistance genes in soil, human gut, and overlapping collection. **(B)** Cross-resistance between AMPs in soil and human gut microbiota. **(C)** Cross-resistance between AMPs in soil microbiota. **(D)** Cross-resistance between AMPs in human gut microbiota.

Furthermore, within the 286 resistance genes identified in both soil and human gut resistomes, 48 of them were recognized as known resistance genes, constituting 16.8% of the total. Notably, when considering the resistomes of soil and the human gut separately, the percentages of known resistance genes were 3.8 and 4.5%, respectively (refer to Figure 4A). This observation indicates that the proportion of known resistance genes among the shared ones in both soil and the human gut is the highest. This finding suggests that shared resistance genes are more likely to represent the authentic resistance genes for microbes, including pathogens, in practical situations.

### 3.5 Cross-resistance is common among AMPs

To confirm the extent of cross-resistance, the resistant colonies identified in the validation phase underwent additional testing. This involved conducting MIC tests on four AMPs not employed in the selection process. Out of the 20 colonies examined, 13 exhibited resistance to at least one AMP that was not part of the selection criteria (refer to Supplementary Table S6). Notably, no collateral sensitivity was observed.

Moreover, when examining resistomes for five antimicrobial peptides found in both soil and the human gut, we observed cross-resistance within a given sample (either soil or the human gut). This phenomenon of cross-resistance also extends across different samples. Particularly noteworthy is the observation that within the human gut microbiota, the extent of cross-resistance is markedly greater compared to that in soil. Furthermore, polymyxin B stands out by demonstrating the highest level of cross-resistance with the other four antimicrobial peptides in both soil and human gut samples (see Figures 4B–D).

In this study, genes displaying resistance to all five antimicrobial peptides are grouped together to form the core resistome. This core resistome comprises 3 resistance genes originating from soil and 45 resistance genes from the human gut, as outlined in Supplementary Table S8. The core resistome encompasses various resistance mechanisms, including membrane modification, protease/peptidase activity, efflux pumps within the transport system, and other mechanisms like cell metabolism and DNA repair. It is worth noting that the members of the core resistome do not coincide with the shared resistance genes found in both soil and the human gut.

### 3.6 Cross-resistance between AMPs and antibiotics is relatively rare

The data from our study was compared to the CARD database. Entries containing antimicrobial peptides in the ontology of antimicrobial drugs were excluded. Consequently, 34 antibiotic resistance genes in the CARD database were identified, demonstrating a high similarity to the antimicrobial peptide resistance genes found in both soil and the human gut (Supplementary Table S9). Among these genes, the top 5 classes of antibiotics exhibiting the greatest cross-resistance with antimicrobial peptides include fluoroquinolones, macrolides, cephalosporins, tetracyclines, and aminoglycosides. The primary resistance mechanism observed is efflux pumps, followed by drug inactivation.

## 4 Discussion

Soil bacteria may possess resistance to nearly all antibiotics (Marshall et al., 2009), and the human gut microbiota acts as a reservoir for resistance to many conventional antibiotics. In our study, we initially focused on whether these two environments also serve as reservoirs for resistance to AMPs. Through metagenomic functional selection against five representative AMPs, we revealed that resistance to AMPs is naturally occurring and widespread in both soil and the human gut. The top 8 meters of soil house approximately 26 × 10^28^ microbes, while the human gastrointestinal tract is inhabited by about 1 × 10^14^ microbes (Whitman et al., 1998; Thursby and Juge, 2017). These microorganisms coexist through the production of diverse AMPs and the development of resistance against various AMPs produced by other microbes.

Consequently, our findings indicate that, in the absence of external AMP contamination, microorganisms in both soil and the human gut can easily acquire low levels of AMP resistance under minimal selection pressure. Additionally, despite the abundant microorganism population in the human gut, it exhibits lower species diversity compared to soil. Furthermore, currently, no AMPs have been approved for treating human gut bacterial infections, including the five peptides used in this study. This may account for the 1.9-fold higher number of resistance genes in the AMP resistome of the soil microbiota compared to the resistome of the human gut microbiota.

Over the last decade, there has been a fluctuating perspective on the difficulty of developing resistance to AMPs. While initial studies indicated that AMP resistance was a formidable challenge (Batoni et al., 2011; Ghosh and Haldar, 2015), subsequent research has demonstrated that AMP resistance can indeed emerge quite readily (Andersson et al., 2016; Kubicek-Sutherland et al., 2017; Spohn et al., 2019). Intriguingly, intricate resistance mechanisms leading to high-level or multidrug resistance can spontaneously arise under specific conditions, such as stepwise selection or exposure to drug gradients (Forsberg et al., 2012; Cullen et al., 2015; Jochumsen et al., 2016; Malik et al., 2017). These seemingly conflicting findings are not inherently contradictory, as the nature of resistance can vary significantly depending on the structural, functional, and physicochemical attributes of the studied AMPs (Spohn et al., 2019). Our study corroborates this notion.

Despite substantial disparities in the species and quantities of microbiota present in soil and the human gut, our findings reveal a similarity in the proportion of resistance gene mechanisms targeting five antimicrobial peptides. This suggests that the composition of the resistome is more closely tied to the antimicrobial peptides used for selection and is not significantly correlated with the microbiota composition in the analyzed samples. Our research affirms that there is a partial intersection between the AMP resistome in soil and the human gut, indicating a sharing of resistance-carrying bacteria between these ecosystems and the potential transfer of resistance. Additionally, specific AMP resistance genes identified in both soil and the human gut exhibit significant similarity to known resistance genes in particular pathogens such as *Klebsiella pneumoniae* and *Pseudomonas aeruginosa*. This suggests the possible exchange or sharing of AMP resistance among environmental sources, humans, and pathogens.

It is worth noting that individuals may encounter microorganisms harboring resistance through various pathways of soil ingestion. These resistant bacteria can act as potential opportunistic pathogens in humans, leading to infections and associated symptoms like vomiting and diarrhea (Marshall et al., 2009). Consequently, resistance may be transferred from soil-derived microorganisms to the human gut microbiota and, in certain conditions, to pathogens, potentially resulting in increased or multidrug resistance (Bialvaei and Samadi, 2015; Colclough et al., 2019).

Notably, the results of our study indicate that the shared resistance genes in soil and the human gut are more likely to drive microbial resistance, including in pathogens. This suggests that bacteria or resistance genes capable of being shared or transferred between soil and human gut microbiota may pose a heightened pathogenic risk.

Although it may seem that the transfer of AMP resistance genes among human gut microorganisms is less common than that of antibiotic resistance genes due to their functional incompatibility with new hosts (Kintses et al., 2019), the results of our study indicate that the increased utilization of AMPs could inevitably hasten the evolution and dissemination of AMP resistance. This is particularly noteworthy for polymyxins (polymyxin B and colistin), which are widely used in clinical settings and exhibited the highest number of resistance genes in our study. Indeed, global studies have demonstrated an escalating prevalence of resistance to polymyxin B (Wang et al., 2018; Kintses et al., 2019), and the mcr-1 plasmid, responsible for conferring colistin resistance, is spreading globally (Bhuiyan et al., 2021).

Resistance to AMPs, whether occurring among AMPs themselves or traditional antibiotics, presents a notable hurdle to the efficacy of AMPs. When cross-resistance emerges between AMPs and human defensins, it can lead to compromised immune function and challenging-to-manage infections (Samuelsen et al., 2005; Habets and Brockhurst, 2012; Fleitas and Franco, 2016). Our research aligns with numerous other studies that underscore the widespread occurrence of cross-resistance among AMPs (Pränting and Andersson, 2010; Forsberg et al., 2012; Blanco et al., 2020; Conceição-Neto et al., 2022). Many of the resistance mechanisms associated with AMPs are linked to bacterial nutrient acquisition and the formation of biofilm matrices. Moreover, the majority of AMP efflux pumps can accept other antimicrobials as substrates, indicating that resistance to AMPs is relatively nonspecific, resulting in common cross-resistance.

Significantly, polymyxin B demonstrates the highest level of cross-resistance with four other AMPs, potentially attributed to its increased clinical use in local hospitals in recent years. The rising concern over polymyxin B resistance stems from the limited availability of effective antimicrobial agents for multidrug-resistant Gram-negative bacterial infections (Kintses et al., 2019; Joseph et al., 2021). Cross-resistance to AMPs is more pronounced in the human gut microbiota compared to soil, potentially due to differences in the types of peptides present in these environments. The human gut microbiota faces the challenge of dealing with host defense peptides, in addition to those produced by other microorganisms. Research indicates that prokaryotic and eukaryotic peptides differ in their spectrum of action, with the former having a narrower target range and the latter exhibiting less specificity, making them effective against a broader range of bacteria (Lázár et al., 2018). This non-specific mechanism elucidates the higher prevalence of cross-resistance in antimicrobial peptides found in the human gut.

A small number of AMP resistance genes that closely resemble known antibiotic resistance genes were identified through our comparison with the Comprehensive Antimicrobial Resistance Database (CARD), indicating the potential for cross-resistance between AMPs and antibiotics. Cross-resistance involves antibiotics such as aminoglycosides (the most common), cephalosporins, tetracyclines, macrolides, and others, as reported in previous studies (Kubicek-Sutherland et al., 2017; Blanco et al., 2020; Conceição-Neto et al., 2022). The clinical treatment options are undeniably narrowed by this limitation. Fortunately, collateral sensitivity to other AMPs or antibiotics may be exhibited by bacteria resistant to certain AMPs or antibiotics (Forsberg et al., 2012; Spohn et al., 2019; Maron et al., 2022). This suggests that the rate of resistance development and cross-resistance could be slowed down by a combination of AMPs or the implementation of a drug-cycling protocol (Aranda et al., 2011; Dobson et al., 2013).

Various bacterial resistance mechanisms to AMPs exist. Besides well-known mechanisms such as LPS modifications, proteases/peptidases, and efflux pumps (Habets and Brockhurst, 2012; Koo and Seo, 2019), the results of our study have revealed additional factors like DNA repair, nitrogen metabolism, and transcriptional regulation that contribute to AMP resistance and cross-resistance. As an illustration, *recA*, a pivotal component in the SOS response and DNA repair, was identified as a core resistome member, conferring resistance to all AMPs examined in our study. The association between *recA* and colistin resistance in *Acinetobacter baumannii* has been documented previously (Alam et al., 2016). Furthermore, *recA* may enhance resistance by facilitating horizontal gene transfer and promoting biofilm formation (Maria-Neto et al., 2012). The results of our study indicate a potential involvement of nitrogen metabolism in AMP resistance, as evidenced by the identification of the gene encoding glutamine synthetase (GS), known as *glnA*, within the core resistome. An example highlighting the connection between nitrogen metabolism and AMP resistance is observed in *E. coli* strains overexpressing GS, which exhibit resistance to the cationic peptide magainin I (Millanao et al., 2020). Additionally, GS is involved in the biosynthesis of the cell wall, maintaining its thickness and the level of crosslinking on peptidoglycan. Inhibition of GS activity may impact the synthesis of peptidoglycan layers, altering bacterial susceptibility (Helmann, 2016). The transcriptional regulator, extracytoplasmic function (ECF) sigma factor *sigW*, was identified as conferring resistance to all AMPs, in our study. While *sigW* is not the direct cause of resistance, it can be activated in response to cell envelope stress, subsequently regulating genes associated with resistance to various AMPs such as nisin, lantibiotics, sublancin, and bacteriocins.^[48]^ Moreover, redox processes, two-component systems, and other mechanisms may also play a role in AMP resistance, but further research is necessary for verification.

The examination of the AMP resistome in soil and the human gut microbiota has revealed key aspects of AMP resistance. These include the likelihood of resistance development and spread, along with the possible contribution of various mechanisms.

## 5 Conclusion

The comprehensive analysis and examination of AMP resistance characteristics and risks from environmental and human gut resistome perspectives were undertaken for the first time in our study. The importance of the microbiota of soil and human gut as sources of the AMP resistome was demonstrated, highlighting the potential transmission of AMP resistance among environments, humans, and pathogens. The potential for the widespread emergence of resistance, facilitated by heightened use of AMPs, was demonstrated through the evolutionary trends observed. Additionally, cross-resistance was found to be common among AMPs, with relative rarity observed between AMPs and antibiotics. This observation suggests the promise of AMPs in combating antibiotic-resistant bacteria, yet their effectiveness in addressing AMP resistance is comparatively limited. Therefore, it is advisable to exercise stringent oversight over the application of AMPs in pertinent domains, particularly given the increasing use of AMPs. This precautionary measure aims to avoid the selection of high-level resistance, particularly in scenarios where there exists the potential for resistance to spread between different environments and humans.

## Data availability statement

The datasets presented in this study can be found in online repositories. The names of the repository/repositories and accession number(s) can be found in the article/Supplementary material.

## Ethics statement

The studies involving humans were approved by the Ethics Committee of The First People’s Hospital of Yunnan Province (KHLL 2021–222). The studies were conducted in accordance with the local legislation and institutional requirements. The participants provided their written informed consent to participate in this study.

## Author contributions

CZ: Data curation, Formal analysis, Writing – original draft. SY: Formal analysis, Writing – review & editing. YL: Data curation, Formal analysis, Writing – review & editing. YS: Conceptualization, Writing – review & editing. XX: Conceptualization, Writing – review & editing.
