# Supplementary material for: Analyzing resistome in soil and Human gut: a study on the characterization and risk evaluation of antimicrobial peptide resistance
Source: Front Microbiol. 2024 Mar 25;15:1352531. doi: 10.3389/fmicb.2024.1352531 (PMC10999558; doi:10.3389/fmicb.2024.1352531)
Supplement: Supplementary file 6 [file Table_6.docx]

Table S6. MICs of retransformed colonies with resistance-conferring plasmids

| **Colony name** | **MIC (μg/ml)** | | | | |
| --- | --- | --- | --- | --- | --- |
|  | GQ26 | SA37 | IB367 | PB | CST |
| pUC118-DH5α | 16 | 16 | 16 | 0.5 | 0.25 |
| pUC118-JM109 | 4 | 8 | 8 | 0.25 | 0.25 |
| pUC118-BL21(DE3) | 8 | 16 | 16 | 1 | 1 |
| pUC118-SGQ1-DH5α | 64 | 32 | 16 | 0.5 | 0.25 |
| pUC118-SGQ1-JM109 | 8 | 16 | 8 | 0.25 | 0.25 |
| pUC118-SGQ1-BL21(DE3) | 16 | 16 | 16 | 1 | 1 |
| pUC118-SGQ2-DH5α | 32 | 32 | 16 | 0.5 | 0.25 |
| pUC118-SGQ2-JM109 | 8 | 16 | 8 | 0.25 | 0.25 |
| pUC118-SGQ2-BL21(DE3) | 16 | 32 | 16 | 1 | 1 |
| pUC118-SSA1-DH5α | 16 | 32 | 32 | 0.5 | 0.5 |
| pUC118-SSA1-JM109 | 4 | 16 | 16 | 0.25 | 0.5 |
| pUC118-SSA1-BL21(DE3) | 8 | 32 | 32 | 1 | 2 |
| pUC118-SSA2-DH5α | 32 | 32 | 32 | 0.5 | 1 |
| pUC118-SSA2-JM109 | 16 | 16 | 16 | 0.25 | 0.25 |
| pUC118-SSA2-BL21(DE3) | 16 | 32 | 16 | 1 | 2 |
| pUC118-SIB1-DH5α | 16 | 16 | 32 | 0.5 | 0.25 |
| pUC118-SIB1-JM109 | 4 | 8 | 32 | 0.25 | 0.25 |
| pUC118-SIB1-BL21(DE3) | 8 | 16 | 32 | 1 | 1 |
| pUC118-SIB2-DH5α | 16 | 16 | 32 | 0.5 | 0.25 |
| pUC118-SIB2-JM109 | 4 | 8 | 16 | 0.25 | 0.25 |
| pUC118-SIB2-BL21(DE3) | 8 | 16 | 32 | 1 | 1 |
| pUC118-SPB1-DH5α | 16 | 16 | 16 | 1 | 0.25 |
| pUC118-SPB1-JM109 | 4 | 8 | 8 | 0.5 | 0.25 |
| pUC118-SPB1-BL21(DE3) | 8 | 16 | 16 | 2 | 1 |
| pUC118-SPB2-DH5α | 16 | 16 | 16 | 2 | 0.5 |
| pUC118-SPB2-JM109 | 4 | 8 | 8 | 0.5 | 0.5 |
| pUC118-SPB2-BL21(DE3) | 8 | 16 | 16 | 2 | 2 |
| pUC118-SCST1-DH5α | 16 | 16 | 16 | 0.5 | 0.5 |
| pUC118-SCST1-JM109 | 4 | 8 | 8 | 0.25 | 0.5 |
| pUC118-SCST1-BL21(DE3) | 8 | 16 | 16 | 1 | 2 |
| pUC118-SCST2-DH5α | 16 | 16 | 16 | 0.5 | 1 |
| pUC118-SCST2-JM109 | 4 | 8 | 8 | 0.25 | 0.5 |
| pUC118-SCST2-BL21(DE3) | 8 | 16 | 16 | 1 | 2 |
| pUC118-FGQ1-DH5α | 32 | 32 | 16 | 1 | 0.5 |
| pUC118-FGQ1-JM109 | 8 | 16 | 8 | 0.5 | 0.5 |
| pUC118-FGQ1-BL21(DE3) | 16 | 32 | 16 | 2 | 2 |
| pUC118-FGQ2-DH5α | 64 | 16 | 32 | 1 | 0.5 |
| pUC118-FGQ2-JM109 | 8 | 8 | 16 | 0.5 | 0.5 |
| pUC118-FGQ2- BL21(DE3) | 16 | 16 | 32 | 2 | 2 |
| pUC118-FSA1-DH5α | 32 | 32 | 16 | 0.5 | 0.5 |
| pUC118-FSA1-JM109 | 16 | 16 | 8 | 0.25 | 2 |
| pUC118-FSA1-BL21(DE3) | 16 | 32 | 16 | 1 | 2 |
| pUC118-FSA2-DH5α | 16 | 32 | 16 | 1 | 0.5 |
| pUC118-FSA2-JM109 | 4 | 16 | 8 | 0.5 | 0.5 |
| pUC118-FSA2-BL21(DE3) | 8 | 32 | 16 | 4 | 2 |
| pUC118-FIB1-DH5α | 16 | 16 | 32 | 0.5 | 0.25 |
| pUC118-FIB1-JM109 | 4 | 8 | 16 | 0.25 | 0.25 |
| pUC118-FIB1-BL21(DE3) | 8 | 16 | 32 | 1 | 1 |
| pUC118-FIB2-DH5α | 16 | 32 | 32 | 1 | 1 |
| pUC118-FIB2-JM109 | 4 | 16 | 32 | 0.5 | 0.5 |
| pUC118-FIB2-BL21(DE3) | 8 | 32 | 32 | 2 | 2 |
| pUC118-FPB1-DH5α | 16 | 16 | 32 | 1 | 2 |
| pUC118-FPB1-JM109 | 4 | 8 | 32 | 0.5 | 1 |
| pUC118-FPB1-BL21(DE3) | 8 | 16 | 32 | 4 | 4 |
| pUC118-FPB2-DH5α | 16 | 16 | 16 | 1 | 1 |
| pUC118-FPB2-JM109 | 4 | 8 | 8 | 0.5 | 0.5 |
| pUC118-FPB2-BL21(DE3) | 8 | 16 | 16 | 2 | 2 |
| pUC118-FCST1-DH5α | 16 | 16 | 16 | 0.5 | 0.5 |
| pUC118-FCST1-JM109 | 4 | 8 | 8 | 0.25 | 0.5 |
| pUC118-FCST1-BL21(DE3) | 8 | 16 | 16 | 1 | 2 |
| pUC118-FCST2-DH5α | 16 | 16 | 16 | 1 | 0.5 |
| pUC118-FCST2-JM109 | 4 | 8 | 8 | 1 | 1 |
| pUC118-FCST2-BL21(DE3) | 8 | 16 | 16 | 2 | 2 |

**Note**: MIC, Minimal inhibitory concentration; GQ26, melittin; SA, cathelicidin-DM; IB, iseganan; PB, polymyxin B; CST, colistin. Description for colony name, ‘pUC118-S(F)GQ1-DH5α’ represents ‘plasmid name-insert of number 1 clone selected against melittin from soil(feces) -*E. coli* DH5α (*E. coli* JM 109, *E. coli* BL21(DE)) strain’.
