# Supplementary material for: Analyzing resistome in soil and Human gut: a study on the characterization and risk evaluation of antimicrobial peptide resistance
Source: Front Microbiol. 2024 Mar 25;15:1352531. doi: 10.3389/fmicb.2024.1352531 (PMC10999558; doi:10.3389/fmicb.2024.1352531)
Supplement: Supplementary file 7 [file Table_7.docx]

Table S7. Known AMP resistance genes identified in soil and human gut microbiota

| Uniprot ID | Gene name | Organism | Pathogen | Sample | Target AMPs in references | Target AMPs in this study |
| --- | --- | --- | --- | --- | --- | --- |
| A7LXW1 | *BACOVA_02677* | *Bacteroides ovatus* | No | F | PB | CST, IB |
| O34951 | *bceR* | *Bacillus subtilis* | No | F | Bacitracin | GQ, IB, PB |
| P21628 | *braE* | *Pseudomonas aeruginosa* | Yes | F | Bacitracin, nisin | PB |
| Q8AA38 | *BT_0627* | *Bacteroides thetaiotaomicron* | Yes | F | PB | SA |
| Q0P9B8 | *cj1136* | *Campylobacter jejuni* | Yes | F | PB | CST, PB, GQ |
| Q18BL7 | *cprB* | *Clostridium difficile* | Yes | F | Nisin, galidermin | CST |
| Q7CPC3 | *cpxA* | *Salmonella typhimurium, Escherichia coli* | Yes | F | Protamine, GQ magainin 2, ApoEdpL-W, PB, | IB, PB |
| A0A8G1KJ80 | *fkpA* | *Klebsiella pneumoniae (ST258)* | Yes | F | CST | CST, IB |
| Q9RQP7 | *icaB* | *Staphylococcus epidermidis* | Yes | F | LL-37, HBD-3 | PB |
| U3Q0D3 | *lgmA* | *Bordetella pertussis* | Yes | F | PB, CST, LL-37, indolicidin, Cp28, HH-C10 | PB |
| Q2TJD0 | *lgtF* | *Campylobacter jejuni* | Yes | F | PB, LL-37 | GQ |
| V5VC90 | *lolD* | *Acinetobacter baumannii* | Yes | F | PB | PB, SA |
| B0VMV4 | *lpxA* | *Acinetobacter baumannii* | Yes | F | PB, CST | PB, CST, GQ, IB |
| B0VMV2 | *lpxD* | *Acinetobacter baumannii* | Yes | F | PB, CST | SA |
| P60611 | *lytR* | *Staphylococcus aureus* | Yes | F | HNP-1, tPMPs | IB, PB |
| Q7BKK4 | *mefE* | *Streptococcus pneumoniae* | Yes | F | LL-37 | IB, PB, SA |
| P52477 | *mexA* | *Pseudomonas aeruginosa* | Yes | F | CST | CST, GQ, IB, PB |
| A0A486TPA3 | *miaA* | *Klebsiella pneumoniae (ST258)* | Yes | F | CST | CST, GQ, IB, SA |
| Q51006 | *mtrE* | *Neisseria meningitidis, Neisseria gonorrhoeae* | Yes | F | PB, LL-37, protegrin-3 | PB, GQ, IB |
| P36556 | *pmrA* | *Salmonella enterica serovar Typhimurium* | Yes | F | PB | GQ |
| Q04873 | *pmrE* | *Salmonella enterica Serovar Typhimurium* | Yes | F | PB | PB, GQ, CST |
| O31054 | *scnF* | *Streptococcus pyogenes* | Yes | F | Streptococcin A-FF22 | SA |
| B2FJW5 | *spgM* | *Stenotrophomonas maltophilia* | Yes | F | PB, CST | SA |
| P69423 | *tatC* | *Salmonella typhimurium* | Yes | F | Protamine, magainin 2, GQ | CST, PB, IB |
| V5VCA9 | *tolR* | *Acinetobacter baumannii* | Yes | F | PB | GQ |
| P39448 | *trkA* | *Vibrio alginolyticus* | Yes | F | PB, Protamine | PB |
| Q9CET4 | *ysaC* | *Lactococcus lactis* | No | F | Bacteriocins, daptomycin, gramicidin | CST, GQ |
| W9B4M6 | *acrB* | *Klebsiella pneumoniae* | Yes | F, S | PB, HNP-1, HBD-1, HBD-2 | CST, PB, GQ |
| Q8Y5F0 | *anrA* | *Listeria monocytogenes* | Yes | F, S | Nisin, bacitracin | CST, PB, SA |
| Q5WNX2 | *bcrD* | *Enterococcus faecalis* | Yes | F, S | Bacitracin | CST, IB, GQ, PB |
| Q8A8D8 | *BT_1229* | *Bacteroides thetaiotaomicron* | Yes | F, S | PB | PB, CST, IB, GQ, SA |
| O67996 | *bvrR* | *Brucella abortus* | Yes | F, S | PB | PB, IB, GQ |
| O68164 | *bvrS* | *Brucella abortus* | Yes | F, S | PB | SA |
| A6L5X9 | *BVU_3469* | *Bacteroides vulgatus* | Yes | F, S | PB | PB, CST, IB |
| R4Z8M6 | *ciaR* | *Group B Streptococcus* | No | F, S | mCRAMP, PB | GQ, SA |
| Q92SV4 | *cmk* | *Sinorhizobium meliloti* | No | F, S | PB | PB, CST, IB, GQ |
| Q18BL2 | *cprA* | *Clostridium difficile* | Yes | F, S | Nisin, galidermin | CST, IB, GQ |
| A6TC03 | *dedA* | *Klebsiella pneumoniae (ST258)* | Yes | F, S | CST | IB, SA |
| A0A8G1KJU9 | *degP* | *Klebsiella pneumoniae (ST258)* | Yes | F, S | CST | GQ |
| P26503 | *exoB* | *Sinorhizobium meliloti* | No | F, S | PB | PB, CST, IB, GQ, SA |
| A0A3N6L3I7 | *ftsX* | *Lactococcus lactis* | No | F, S | Bacteriocins, daptomycin, and gramicidin | PB, CST, IB, GQ |
| A0A4V6DW09 | *galT* | *Campylobacter jejuni* | Yes | F, S | PB | PB, CST |
| B2HHR7 | *kasB* | *Mycobacterium tuberculosis, Mycobacterium marinum* | Yes | F, S | Human defensins | PB, IB, GQ, SA |
| Q9R9N2 | *lpsB* | *Sinorhizobium meliloti* | No | F, S | PB | CST, GQ, SA |
| P9WFU7 | *lysX* | *Mycobacterium tuberculosis* | Yes | F, S | HNP1-3 | PB, CST, IB, GQ, SA |
| P52002 | *mexB* | *Pseudomonas aeruginosa* | Yes | F, S | CST | PB, CST, IB, GQ, SA |
| O34442 | *mgtE* | *Bacillus subtilis* | No | F, S | PB | PB, CST, IB, GQ |
| Q9RC27 | *mrsF* | *Bacillus sp. (strain HIL-Y85/54728)* | No | F, S | Mersacidin | CST, IB, GQ, SA |
| Q9KQW9 | *msbA* | *Vibrio cholerae* | Yes | F, S | PB | PB, CST, IB, GQ, SA |
| X5ESJ1 | *mtrD* | *Neisseria meningitidis, Neisseria gonorrhoeae* | Yes | F, S | PB, LL-37, protegrin-2 | PB, CST, SA |
| A0A0E1U924 | *mucD* | *Burkholderia genus* | No | F, S | PB | PB, CST, IB, SA |
| Q52990 | *phoB* | *Sinorhizobium meliloti* | No | F, S | PB | PB, CST, IB, GQ, SA |
| Q92SA5 | *phoR* | *Sinorhizobium meliloti* | No | F, S | PB | PB, CST, IB |
| Q04707 | *ponA* | *Streptococcus pneumoniae* | Yes | F, S | HNP-1, LL-37 | CST, IB, SA |
| P58662 | *rcsC* | *Pathogenic or commensal Enterobacteriaceae species* | Yes | F, S | PB | PB, CST, IB, GQ, SA |
| O54068 | *rkpK* | *Sinorhizobium meliloti* | No | F, S | PB | PB, CST, IB, SA |
| Q56876 | *rosB* | *Yersinia sp.* | Yes | F, S | PB | CST, GQ, SA |
| A0A7U4CHE4 | *rpoA* | *Staphylococcus aureus* | Yes | F, S | Pexiganan | PB, IB |
| O08352 | *rpoN* | *Listeria monocytogenes* | Yes | F, S | Mesentericin Y105 | CST, SA |
| Q9I5U5 | *rsmA* | *Pseudomonas aeruginosa* | Yes | F, S | PB, CST | PB, CST, SA |
| Q2FK78 | *SAUSA300_0186* | *Staphylococcus aureus (strain USA300)* | Yes | F, S | LL-37 | PB, IB, SA, GQ |
| A0A0H2XI70 | *SAUSA300_0988* | *Staphylococcus aureus (strain USA300)* | Yes | F, S | LL-37 | CST, GQ |
| A0A0H2XGX1 | *SAUSA300_1036* | *Staphylococcus aureus (strain USA300)* | Yes | F, S | LL-37 | PB, CST, IB, GQ |
| Q2FHU3 | *SAUSA300_1037* | *Staphylococcus aureus (strain USA300)* | Yes | F, S | LL-37 | CST |
| Q2FH23 | *SAUSA300_1308* | *Staphylococcus aureus (strain USA300)* | Yes | F, S | LL-37 | CST, GQ |
| A0A0H2XGS0 | *SAUSA300_1336* | *Staphylococcus aureus (strain USA300)* | Yes | F, S | LL-37 | CST, SA |
| Q09II0 | *sboF* | *Streptococcus salivarius* | No | F, S | Salivaricin B | PB, IB |
| A0A0H2UPL0 | *SP_0912* | *Streptococcus pneumoniae* | Yes | F, S | Bacitracin, lincomycin, nisin | PB, GQ, SA |
| E0U280 | *spaF* | *Bacillus subtilis* | No | F, S | Subtilin | PB, GQ, SA |
| Q8DQF8 | *spr0694* | *Streptococcus pneumoniae* | Yes | F, S | LL-37, nisin, bacitracin | PB, CST, IB, GQ, SA |
| Q8ZQT5 | *tolB* | *Salmonella enterica serovar Typhimurium* | Yes | F, S | PB | PB, CST, IB, SA |
| Q8ZNJ9 | *yejE* | *Salmonella enterica serovar Typhimurium* | Yes | F, S | Protamine, PB, GQ, HBD-1,HBD-4 | PB, IB, GQ, SA |
| Q8ZNJ8 | *yejF* | *Salmonella enterica serovar Typhimurium* | Yes | F, S | Protamine, PB, GQ, HBD-1,HBD-4 | CST, IB, GQ |
| Q8ZNF3 | *yfbE* | *Salmonella Typhimurium* | Yes | F, S | PB | PB, CST, IB, GQ, SA |
| A0A1Z1SYP0 | *acrA* | *Proteus mirabilis* | Yes | S | PB | IB, GQ |
| P33772 | *amiA* | *Salmonella typhimurium, Escherichia coli* | Yes | S | Protamine, LL37, magainin-2, GQ, HNP-1, | SA |
| Q8RKC0 | *as-48H* | *Enterococcus faecalis* | Yes | S | AS-48 | GQ |
| A0A8G1NS55 | *bamB* | *Klebsiella pneumoniae (ST258)* | Yes | S | CST | GQ, SA |
| O34697 | *bceA* | *Bacillus subtilis* | No | S | Bacitracin, plectasin, mersacidin, actagardine | CST |
| Q51693 | *capD* | *Staphylococcus epidermidis* | Yes | S | LL-37, HBD-3 | PB, IB |
| Q93SN1 | *colR* | *Pseudomonas aeruginosa* | Yes | S | PB | CST |
| Q9HZD1 | *cprR* | *Pseudomonas aeruginosa* | Yes | S | PB | GQ |
| Q92U93 | *ddhA* | *Sinorhizobium meliloti* | No | S | PB | PB |
| Q9X2N5 | *dltB* | *Staphylococcus aureus, Staphylococcus xylosus* | Yes | S | HNP-1, HNP-3, protegrins, nisin tachyplesins, magainin II, gallidermin | SA |
| P39577 | *dltE* | *Bacillus subtilis* | No | S | Lysozyme, lantibiotic | CST |
| Q2FD54 | *emrB* | *Acinetobacter baumannii* | Yes | S | CST | PB, CST |
| Q54002 | *epiF* | *Staphylococcus epidermidis* | Yes | S | Epidermin, gallidermin | CST |
| P38134 | *etk* | *Escherichia coli, Salmonella sp., Pseudomonas aeruginosa* | Yes | S | PB | CST |
| P33699 | *exoT* | *Sinorhizobium meliloti* | No | S | PB | IB |
| Q92R91 | *feuP* | *Sinorhizobium meliloti* | No | S | PB | IB, SA |
| P45602 | *galE* | *Klebsiella pneumoniae (ST258)* | Yes | S | CST | GQ |
| P0C1R9 | *hemB* | *Staphylococcus aureus* | Yes | S | LL-37, HBD-2, HBD-3, CST, lactoferricinB, Protamine | PB, CST, IB, GQ, SA |
| B4EB35 | *hldA* | *Burkholderia cenocepacia* | Yes | S | PB, GQ, HNP | SA |
| B4EB34 | *hldD* | *Burkholderia cenocepacia* | Yes | S | GQ, PB, HNP | GQ |
| A0A7X1HSH8 | *hupA* | *Klebsiella pneumoniae (ST258)* | Yes | S | CST | CST |
| O32197 | *liaR* | *Bacillus subtilis* | No | S | Daptomycin, LL-37, HBD-3, nisin, gallidermin, mersacidin, friulimicin | PB, CST, GQ, SA |
| Q9R9N1 | *lpsE* | *Sinorhizobium meliloti* | No | S | PB | SA |
| B8Y3V5 | *lpxL1* | *Neisseria meningitidis* | Yes | S | PB | IB |
| E0TUX7 | *ltaSa* | *Bacillus subtilis W168* | No | S | Nisin | CST |
| A0A0H3JZF2 | *lysC* | *Staphylococcus aureus* | Yes | S | CAP18 and hBD3 | CST, GQ, SA |
| Q53705 | *lytS* | *Staphylococcus aureus* | Yes | S | HNP-1 and tPMPs | PB, GQ, SA |
| P75831 | *macB* | *Escherichia coli* | Yes | S | Bacitracin, CST | PB, IB, GQ, SA |
| Q8VUH2 | *mbrA* | *Streptococcus mutans* | Yes | S | Bacitracin | CST, GQ |
| P36640 | *mgtA* | *Salmonella* | Yes | S | PB | GQ |
| A0A2X3ERP0 | *mrcB* | *Klebsiella pneumoniae (ST258)* | Yes | S | CST | CST |
| Q48597 | *nisF* | *Lactococcus lactis* | No | S | Nisin | CST |
| Q9I2U4 | *parS* | *Pseudomonas aeruginosa* | Yes | S | PB | SA |
| O31773 | *pbpX* | *Bacillus subtilis* | No | S | Lysozyme | SA |
| O34798 | *pdaC* | *Bacillus subtilis* | No | S | Lysozyme | CST |
| A0A0E0UU11 | *pgdA* | *Listeria monocytogenes, Enterococcus faecalis* | Yes | S | Lysozyme | CST |
| A0A378FSX7 | *Pgi* | *Klebsiella pneumoniae (ST258)* | Yes | S | CST | PB |
| Q5E6N5 | *pgm* | *Aliivibrio fischeri (Vibrio fischeri)* | Yes | S | PB | CST |
| A0A376GSU7 | *pgsA2* | *Corynebacterium striatum* | No | S | Daptomycin | PB, CST |
| G9JL97 | *phoP* | *Pseudomonas aeruginosa, Salmonella typhimurium, Escherichia coli* | Yes | S | PB, protamine, defensins | PB |
| Q52989 | *phoU* | *Sinorhizobium meliloti* | No | S | PB | CST, IB, GQ, SA |
| W8UWD7 | *pmtR* | *Staphylococcus aureus* | Yes | S | GQ | PB, IB, SA |
| O06980 | *psdA* | *Bacillus subtilis* | No | S | Nisin, galidermin | CST |
| Q92SA1 | *pstB* | *Sinorhizobium meliloti* | No | S | PB | CST |
| P24555 | *ptrB* | *Escherichia coli MS 79-10* | Yes | S | PGLa | PB, IB |
| F3QP53 | *qseB* | *Parasutterella excrementihominis* | No | S | PB | PB |
| O82876 | *rgpD* | *Streptococcus mutans* | Yes | S | Bacitracin | CST |
| Q56877 | *rosA* | *Yersinia sp.* | Yes | S | PB | CST |
| B4EWL1 | *sapD* | *Proteus mirabilis* | Yes | S | GQ, protamine | PB, CST, SA |
| Q2FHN4 | *SAUSA300_1097* | *Staphylococcus aureus (strain USA300)* | Yes | S | LL-37 | SA |
| Q2FHA3 | *SAUSA300_1228* | *Staphylococcus aureus (strain USA300)* | Yes | S | LL-37 | CST |
| A0A0H2XEF1 | *SAUSA300_1465* | *Staphylococcus aureus (strain USA300)* | Yes | S | LL-37 | PB, CST, IB, GQ |
| Q2FGL3 | *SAUSA300_1469* | *Staphylococcus aureus (strain USA300)* | Yes | S | LL-37 | PB |
| A0A0H2XI43 | *SAUSA300_1515* | *Staphylococcus aureus (strain USA300)* | Yes | S | LL-37 | CST |
| A0A0H2XGC9 | *SAUSA300_1865* | *Staphylococcus aureus (strain USA300)* | Yes | S | LL-37 | SA |
| Q92R88 | *SMc02366* | *Sinorhizobium meliloti* | No | S | PB | CST, SA |
| Q92R87 | *SMc02367* | *Sinorhizobium meliloti* | No | S | PB | CST |
| Q92LN9 | *SMc03097* | *Sinorhizobium meliloti* | No | S | PB | SA |
| Q8DUP1 | *SMU.863* | *Streptococcus mutans* | Yes | S | Bacitracin | PB, CST, IB |
| Q8DUP0 | *SMU.864* | *Streptococcus mutans* | Yes | S | Bacitracin | PB, IB, GQ SA |
| E0U278 | *spaG* | *Bacillus subtilis* | No | S | Subtilin | CST |
| O34525 | *sppA* | *Bacillus subtilis W168* | No | S | Nisin | PB |
| Q8DQ77 | *spr0812* | *Streptococcus pneumoniae* | Yes | S | Bacitracin, vancoresmycin | CST |
| B4ED80 | *suhB* | *Burkholderia cenocepacia* | Yes | S | CST | CST, GQ |
| A0A377WEW3 | *tagA* | *Klebsiella pneumoniae (ST258)* | Yes | S | CST | CST, GQ |
| A0A1Z1SRV5 | *tolC* | *Proteus mirabilis* | Yes | S | PB | PB |
| Q9RL74 | *vraD* | *Staphylococcus aureus* | Yes | S | Bacitracin, nisin | PB, IB, GQ, SA |
| Q7A2Q1 | *vraR* | *Staphylococcus aureus* | Yes | S | Vancomycin, daptomycin, ovispirin-1, bacitracin | PB, CST, IB, GQ, SA |
| Q8ZNK0 | *yejA* | *Salmonella enterica serovar Typhimurium* | Yes | S | GQ, PB, Protamine, HBD-1, HBD-2 | GQ, CST |
| Q7CQ74 | *yejB* | *Salmonella enterica serovar Typhimurium* | Yes | S | GQ, PB, Protamine, HBD-1, HBD-2 | SA |
| O52325 | *yfbG* | *Salmonella typhimurium* | Yes | S | PB | CST |
| Q11137 | *zapA* | *Proteus mirabilis* | Yes | S | LL37, HBD-1 | GQ, SA |

**Note:** UniProt ID, ID of the known resistance gene in UniProt; Gene name, name of the associated gene; Organism：species in which the gene conferred resistance; Pathogen：whether the known genes come frome pathogens. Sample, where the similar gene in this study came from.; Target AMPs in references : target AMPs for resistance genes in previous references. Target AMPs in this study: target AMPs for resistance genes in this study. F for feces (human gut) and S for soil.
