# Supplementary material for: Analyzing resistome in soil and Human gut: a study on the characterization and risk evaluation of antimicrobial peptide resistance
Source: Front Microbiol. 2024 Mar 25;15:1352531. doi: 10.3389/fmicb.2024.1352531 (PMC10999558; doi:10.3389/fmicb.2024.1352531)
Supplement: Supplementary file 8 [file Table_8.docx]

Table S8. Core resistome of antimicrobial peptides in soil and human gut microbiota

| Uniprot ID | Resistance mechanism | Gene name | Organism | Sample |
| --- | --- | --- | --- | --- |
| P44468 | Membrane modification | mrdB | Haemophilus influenzae | S |
| O31526 | Membrane modification | yesW | Bacillus subtilis | F |
| P0AD20 | Membrane modification | yohK | Shigella flexneri | F |
| P18159 | Membrane modification | pgcA | Bacillus subtilis | F |
| A6LD45 | Protease | lon | Parabacteroides distasonis | F |
| O34666 | Protease | ctpA | Bacillus subtilis | F |
| A6L2J8 | Peptidase | dpp7 | Phocaeicola vulgatus | F |
| O06984 | Transport system | yvdB | Bacillus subtilis | F |
| O05218 | Other | ywrD | Bacillus subtilis | S |
| P24559 | Other | pilT | Pseudomonas aeruginosa | S |
| O05519 | Other | ydiF | Bacillus subtilis | F |
| P22106 | Other | asnB | Escherichia coli | F |
| A6L2N4 | Other | prmA | Phocaeicola vulgatus | F |
| P37454 | Other | exoA | Bacillus subtilis | F |
| A0A2I9 | Other | undec1A | Unknown prokaryotic organism | F |
| A6L4C6 | Other | fbp | Phocaeicola vulgatus | F |
| P18775 | Other | dmsA | Escherichia coli | F |
| Q68BJ6 | Other | TK1108 | Thermococcus kodakarensis | F |
| A6L3H3 | Other | rnz | Phocaeicola vulgatus | F |
| A6KZJ9 | Other | truB | Phocaeicola vulgatus | F |
| T2KMF4 | Other | BN863_21930 | Formosa agariphila | F |
| Q8FTH8 | Other | thiED | Corynebacterium efficiens | F |
| O86365 | Other | Rv0584 | Mycobacterium tuberculosis | F |
| A6L0E8 | Other | gltX | Phocaeicola vulgatus | F |
| O34872 | Other | ytiB | Bacillus subtilis | F |
| P49008 | Other | nahA | Porphyromonas gingivalis | F |
| Q89YY3 | Other | clpB | Bacteroides thetaiotaomicron | F |
| L7P9J4 | Other | IL45_01505 | Nonlabens ulvanivorans | F |
| A7BFV7 | Other | spt | Sphingobacterium spiritivorum | F |
| T2KPJ3 | Other | BN863_21920 | Formosa agariphila | F |
| A6KZR2 | Other | coaD | Phocaeicola vulgatus | F |
| C0ZBR4 | Other | rocD | Brevibacillus brevis | F |
| P15623 | Other | glnA | Bacteroides fragilis | F |
| P59199 | Other | polA | Streptococcus pneumoniae serotype 4 | F |
| Q45585 | Other | sigW | Bacillus subtilis | F |
| P39823 | Other | pssA | Bacillus subtilis | F |
| Q8L7W8 | Other | FUC95A | Arabidopsis thaliana | F |
| E0D7H5 | Other | bsc3 | Alternaria brassicicola | F |
| P0AGF4 | Other | xylE | Escherichia coli (strain K12) | F |
| P94316 | Other | gdhB | Bacteroides fragilis | F |
| A6L7P7 | Other | pgi | Phocaeicola vulgatus | F |
| Q59717 | Other | recA | Prevotella ruminicola | F |
| A6L404 | Other | purT | Phocaeicola vulgatus | F |
| Q9KCZ4 | Other | glcK | Alkalihalobacillus halodurans | F |
| A5FZB9 | Other | hutH | Acidiphilium cryptum | F |
| P0DPE4 | Other | N646_1023 | Vibrio alginolyticus | F |
| O83668 | Other | fba | Treponema pallidum | F |
| Q8A1G1 | Other | susC | Bacteroides thetaiotaomicron | F |

**Note:** UniProt ID, ID of the annotated gene in UniProt; Gene name, name of the annotated gene; Resistance mechanism, description of resistance mechanism based on UniProt; Organism, organism in which the gene conferred resistance; Sample, where the annotated gene came from in our study. F for feces (human gut) and S for soil.
