# Supplementary material for: Analyzing resistome in soil and Human gut: a study on the characterization and risk evaluation of antimicrobial peptide resistance
Source: Front Microbiol. 2024 Mar 25;15:1352531. doi: 10.3389/fmicb.2024.1352531 (PMC10999558; doi:10.3389/fmicb.2024.1352531)
Supplement: Supplementary file 9 [file Table_9.docx]

Table S9. Antibiotic resistance genes identified from AMP resistome in soil and human gut

| **ARO accession** | **ARO name** | **Drug class** | **Resistance mechanism** | **Sample** |
| --- | --- | --- | --- | --- |
| 3000793 | mdtB | aminocoumarin antibiotic | antibiotic efflux | S |
| 3002522 | novA | aminocoumarin antibiotic | antibiotic efflux | S |
| 3000828 | baeR | aminocoumarin antibiotic; aminoglycoside antibiotic | antibiotic efflux | S |
| 3005090 | RanB | aminoglycoside antibiotic | antibiotic efflux | F |
| 3005091 | RanA | aminoglycoside antibiotic | antibiotic efflux | F |
| 3004144 | AxyY | aminoglycoside antibiotic; cephalosporin; fluoroquinolone antibiotic; macrolide antibiotic | antibiotic efflux | F |
| 3003010 | ceoB | aminoglycoside antibiotic; fluoroquinolone antibiotic | antibiotic efflux | F |
| 3003063 | ykkC | aminoglycoside antibiotic; phenicol antibiotic; tetracycline antibiotic | antibiotic efflux | S |
| 3000504 | golS | carbapenem; cephalosporin; cephamycin; monobactam; penam; penem; phenicol antibiotic | antibiotic efflux | S |
| 3000216 | acrB | cephalosporin; disinfecting agents and antiseptics; fluoroquinolone antibiotic; glycylcycline; penam; phenicol antibiotic; rifamycin antibiotic; tetracycline antibiotic | antibiotic efflux | S |
| 3004042 | Eclo_acrA | cephalosporin; disinfecting agents and antiseptics; fluoroquinolone antibiotic; glycylcycline; penam; phenicol antibiotic; rifamycin antibiotic; tetracycline antibiotic | antibiotic efflux | S |
| 3000784 | cmeB | cephalosporin; fluoroquinolone antibiotic; fusidane antibiotic; macrolide antibiotic | antibiotic efflux | F |
| 3000838 | arlR | disinfecting agents and antiseptics; fluoroquinolone antibiotic | antibiotic efflux | F, S |
| 3000808 | MexI | disinfecting agents and antiseptics; fluoroquinolone antibiotic; tetracycline antibiotic | antibiotic efflux | F |
| 3004103 | QepA2 | fluoroquinolone antibiotic | antibiotic efflux | S |
| 3003948 | efrA | fluoroquinolone antibiotic; macrolide antibiotic; rifamycin antibiotic | antibiotic efflux | F |
| 3000777 | adeF | fluoroquinolone antibiotic; tetracycline antibiotic | antibiotic efflux | S |
| 3004039 | Ecol_emrE | macrolide antibiotic | antibiotic efflux | S |
| 3000535 | macB | macrolide antibiotic | antibiotic efflux | F, S |
| 3000816 | mtrA | macrolide antibiotic; penam | antibiotic efflux | S |
| 3003950 | msbA | nitroimidazole antibiotic | antibiotic efflux | F, S |
| 3003705 | mexN | phenicol antibiotic | antibiotic efflux | F |
| 3003986 | TaeA | pleuromutilin antibiotic | antibiotic efflux | F, S |
| 3004033 | tetB(46) | tetracycline antibiotic | antibiotic efflux | S |
| 3002644 | APH(3')-IIa | aminoglycoside antibiotic | antibiotic inactivation | F |
| 3006226 | CepA-49 | cephalosporin | antibiotic inactivation | F |
| 3000979 | TEM-116 | cephalosporin; monobactam; penam; penem | antibiotic inactivation | F, S |
| 3002513 | LRA-19 | cephalosporin; penam | antibiotic inactivation | S |
| 3000498 | ErmF | lincosamide antibiotic; macrolide antibiotic; streptogramin A antibiotic; streptogramin B antibiotic; streptogramin antibiotic | antibiotic target alteration | F |
| 3000616 | mel | macrolide antibiotic; streptogramin antibiotic | antibiotic target protection | F |
| 3003746 | optrA | oxazolidinone antibiotic; phenicol antibiotic | antibiotic target protection | F |
| 3000191 | tet(Q) | tetracycline antibiotic | antibiotic target protection | F |
| 3003105 | dfrA3 | diaminopyrimidine antibiotic | antibiotic target replacement | F |
| 3004361 | sul4 | sulfonamide antibiotic | antibiotic target replacement | S |

**Note:** AMP, antimicrobial peptide; CARD, Comprehensive Antibiotic Resistance Database; ARO accession, Antibiotic Resistance Ontology Accession in CARD; Drug class, drugs that the gene confers resistance to; Resistance mechanism, functional category of the resistance according to functional annotation in CARD; Sample, where the AMP resistance gene came from. F for feces (human gut) and S for soil.
